# Simultaneous Occurrence of Guillain-Barré Syndrome in Three Members of the Same Family: A Case Report

**DOI:** 10.7759/cureus.29356

**Published:** 2022-09-20

**Authors:** Assam Ullah, Salman Khan, Omama Humayun, Sana Ullah, Noor Fatima

**Affiliations:** 1 Internal Medicine, Khyber Teaching Hospital, Peshawar, PAK; 2 Department of Medicine, Lady Reading Hospital, Peshawar, PAK; 3 Department of Medicine, Fatima Memorial Hospital College of Medicine and Dentistry, Lahore, PAK

**Keywords:** sibling, autoimmune, genetic susceptibility, ascending paralysis, aman, familial, guillain-barré syndrome

## Abstract

Guillain-Barré syndrome (GBS) is an autoimmune demyelinating disease that is usually triggered by an antecedent infection and is characterized by flaccid paralysis and hyporeflexia. Although a sporadic disease, a few cases of GBS have been reported in families, suggesting a genetic predisposition. Here, we present a case of simultaneous occurrence of GBS in three members of the same family, with two members having a preceding history of diarrhea. They were diagnosed via cerebrospinal fluid routine examination and nerve conduction study and responded to plasmapheresis. This suggests possible genetic susceptibility along with environmental triggers in the pathogenesis of GBS.

## Introduction

Guillain-Barré syndrome (GBS) is one of the causes of progressive flaccid paralysis which is often provoked by a preceding infection. GBS occurs worldwide with an incidence of 1-2 cases per 100,000 per year [1,2]. Most cases of acute polyneuropathy of GBS occur when an immune response directed against an antecedent infection cross-reacts with epitopes on peripheral nerves, leading to demyelination and subsequent weakness [2]. Although GBS is a sporadic disease, rare familial cases have been reported, making it a complex genetic disorder affected by environmental and genetic factors [3].

Here, we describe three cases of definite GBS in a single generation of a family that presented simultaneously to the hospital.

## Case presentation

Case one

A 43-year-old woman presented with a two-week history of weakness involving both upper and lower limbs, preceded by diarrhea. She started developing weakness in both lower limbs, described as heaviness and a dragging feeling in the lower limbs with subsequent development of weakness in the distal upper arms. On examination, she was afebrile, alert, and conscious. Her pulse rate was 80 beats per minute, blood pressure was 120/70 mmHg, respiratory rate was 16 breaths per minute, and oxygen saturation was 95% on room air. Neurological examination revealed a Medical Research Council (MRC) power grading of 2/5 in proximal while 1/5 in distal muscles of upper and lower limbs. Deep tendon reflexes were absent in lower limbs and 1+ in upper limbs. The rest of the neurological examination was unremarkable. On investigations, a complete blood picture showed hemoglobin of 10.5 g/dL, mean corpuscular volume (MCV) of 76 fL, white blood cell (WBC) count of 10,300/cm^3^, and platelets of 2,56,000/cm^3^. Stool cultures were negative, and electrocardiogram (ECG), thyroid function tests, and metabolic profile revealed no abnormalities. Cerebrospinal fluid (CSF) examination showed normal cells and proteins (Table 1). She was diagnosed as a case of GBS based on the nerve conduction study (NCS), which suggested acute motor axonal neuropathy (AMAN) variant of GBS. She received four sessions of plasmapheresis every 48 hours and responded gradually to treatment with a power of 4/5 in both upper and lower limbs at the time of discharge from the hospital.

**Table 1:** Cerebrospinal fluid findings. CSF = cerebrospinal fluid; WBC = white blood cell; RBC = red blood cell

CSF examination	Reference range	Case 1	Case 2	Case 3
WBC (/cm^3^)	0–5	<5	<5	3
RBC (/cm^3^)	0–10	10	50	15
Glucose (mg/dL)	50–75	68	56	76
Protein (mg/dL)	20–40	25.6	33.4	33

Case two

A 40-year-old woman, younger sister to the first patient, presented with weakness involving both upper and lower limbs for two weeks. She had no history of diarrhea and upper respiratory or viral infection. She started developing weakness in both lower limbs initially with a tingling sensation and eventually was unable to walk or stand unsupported with frequent falls. Subsequently, she also started developing weakness and a tingling sensation in the distal upper arms. On physical examination, she was alert, conscious, and oriented to time, place, and person. She had a blood pressure of 140/90 mmHg, pulse rate of 90 beats per minute, respiratory rate of 18 breaths per minute with a normal breathing pattern, and a temperature of 98.6°F. On neurological examination, MRC power grading of 3/5 in proximal while 1/5 in distal muscles of both upper and lower limbs was noted. Plantar reflexes were flexors bilaterally, and cranial nerves were intact. Deep tendon reflexes were absent in lower limbs and 2+ in upper limbs. On investigations, a complete blood picture showed hemoglobin of 12.3 g/dL, MCV of 78 fL, WBC count of 9,260/cm^3^, and platelet count of 2,15,000/cm^3^. She had no abnormalities on ECG, and a metabolic profile showed normal serum electrolytes, renal function tests, and liver function tests. Thyroid function tests were normal and stool cultures were negative. CSF examination was unremarkable (Table 1). She was also diagnosed as a case of GBS based on NCS which suggested the GBS variant AMAN. She, like her sister, received four sessions of plasmapheresis every 48 hours, and at the time of discharge from the hospital, her power was 4/5 in both upper and lower limbs.

Case three

A 50-year-old man, husband to the younger sister and first cousin to both sisters, presented with a two-week history of weakness involving lower limbs. He also had a history of diarrhea preceding the weakness one month back. He had no weakness in the upper limbs. On physical examination, he was alert, conscious, and oriented to time place, and person. On examination, he had a blood pressure of 120/90 mmHg, pulse rate of 88 beats per minute, respiratory rate of 17 breaths per minute with a normal breathing pattern, and a temperature of 98.6°F. MRC power grading of 1/5 was observed in all muscle groups of both lower limbs. Plantar reflexes were flexors bilaterally, and cranial nerves were intact. Deep tendon reflexes were 1+ in lower limbs and 2+ in upper limbs. On investigations, a complete blood picture showed hemoglobin of 13.5 g/dL, MCV of 78.2 fL, WBC count of 6,320/cm^3^, and platelet count of 2,11,000/cm^3^. The erythrocyte sedimentation rate was 24 mm/first hour, and thyroid function tests showed no abnormalities. ECG was normal, and a metabolic profile showed normal serum electrolytes, renal function tests, and liver function tests. Stool cultures turned out to be negative. CSF examination showed WBCs 3/cm^3^, RBCs 15/cm^3^, glucose 76 mg/dL, and proteins 33/dL (Table 1). He was also diagnosed as a case of GBS based on NCS which suggested the GBS variant AMAN. He, like his wife and her sister, received four sessions of plasmapheresis every 48 hours and responded gradually to treatment with power returning to normal upon discharge.

## Discussion

GBS is an autoimmune disease affecting peripheral nerves, leading to weakness, reduced reflexes, and ascending paralysis occurring in the legs, arms, and even respiratory and facial muscles. It is a heterogeneous syndrome with several variant forms, such as acute inflammatory demyelinating polyneuropathy (AIDP), AMAN, and acute motor and sensory axonal neuropathy (AMSAN) [2]. The etiologic triggers of GBS can be environmental or genetic. Infection with *Campylobacter jejuni*, which is a leading cause of acute gastroenteritis, is the most common antecedent which triggers GBS [4]. Other antecedent infectious triggers include cytomegalovirus, influenza A and B, human immunodeficiency virus, severe acute respiratory syndrome coronavirus 2, Zika virus, Epstein-Barr virus, herpes simplex virus, etc.

Although the genetic basis of GBS remains uncertain, previous research has revealed associations between some genes and the incidence of GBS [5]. In a report to establish the genetic etiology of GBS, 12 families, of which at least two members developed GBS, were included. In this study, the more frequent occurrence of GBS within siblings, the earlier onset of GBS in successive generations, and the patient having GBS four times might point toward a genetic susceptibility [3]. A case report revealed that three of five children who were born to consanguineous parents suffered from GBS before the age of three years, which suggests that there may be a genetic component in the pathogenesis [6]. Some studies have suggested that GBS has a possible association with intercellular adhesion molecule-1 (ICAM1), interleukin-17 (IL-17), signal transducer and activator of transcription 3 (STAT3), several single-nucleotide polymorphisms (SNPs), human leukocyte antigens (HLA) genes, and tumor necrosis factor-alpha (TNFα) [5,7,8].

We report three members of the same family who presented with weakness and were ultimately diagnosed as the AMAN variant of GBS based on NCS and clinical features. CSF analysis revealed no albuminocytologic dissociation, and the simultaneous presentation of all three patients in the same family with the same variant was quite unusual, unlike the previous case report which reported the occurrence of GBS in four siblings years apart [9]. Two out of the three patients in our case had a preceding history of diarrhea, while the other patient reported no history of diarrhea or respiratory tract infection. This points to the significance of environmental factors along with possible genetic predisposition in these patients. Outbreaks of GBS attributed to *C. jejuni* have also been reported in Peru, United States, and Mexico [10,11]. However, in this case, no increased incidence of GBS was reported in the area, and the stool cultures in our cases were negative.

The aforementioned evidence suggests that genetic factors may have a role in the still uncertain etiology of this disorder.

## Conclusions

GBS is usually considered a sporadic autoimmune disease affecting nerves, usually preceded by an infection. However, a few familial cases have been reported, suggesting genetic susceptibility. The presence of antecedent triggers in familial cases suggests a complex etiology of GBS. Further molecular and genetic studies need to be conducted in large population groups to determine the unexpected occurrence of familial cases in an otherwise known sporadic illness.
